# Transient Receptor Potential Ankyrin-1 (TRPA1) Block Protects against Loss of White Matter Function during Ischaemia in the Mouse Optic Nerve

**DOI:** 10.3390/ph14090909

**Published:** 2021-09-09

**Authors:** Wendy Lajoso, Grace Flower, Vincenzo Giacco, Anjuli Kaul, Circe La Mache, Andra Brăban, Angela Roxas, Nicola B. Hamilton

**Affiliations:** Wolfson Centre for Age-Related Diseases, Institute of Psychiatry, Psychology and Neuroscience, Guy’s Campus, King’s College London, London SE1 1UL, UK; lajoso.wendy@gmail.com (W.L.); grace.flower@kcl.ac.uk (G.F.); Vincenzo.giacco@kcl.ac.uk (V.G.); anjuli.1.kaul@kcl.ac.uk (A.K.); circelamache@gmail.com (C.L.M.); andra.braban@kcl.ac.uk (A.B.); angela.roxas@kcl.ac.uk (A.R.)

**Keywords:** transient receptor potential ankyrin-1 (TRPA1), oxygen and glucose deprivation, ischaemia, oligodendrocytes, optic nerve, myelin, compound action potential

## Abstract

Oligodendrocytes produce myelin, which provides insulation to axons and speeds up neuronal transmission. In ischaemic conditions, myelin is damaged, resulting in mental and physical disabilities. Recent evidence suggests that oligodendrocyte damage during ischaemia can be mediated by Transient Receptor Potential Ankyrin-1 (TRPA1), whose activation raises intracellular Ca^2+^ concentrations and damages compact myelin. Here, we show that TRPA1 is constitutively active in oligodendrocytes and the optic nerve, as the specific TRPA1 antagonist, A-967079, decreases basal oligodendrocyte Ca^2+^ concentrations and increases the size of the compound action potential (CAP). Conversely, TRPA1 agonists reduce the size of the optic nerve CAP in an A-967079-sensitive manner. These results indicate that glial TRPA1 regulates neuronal excitability in the white matter under physiological as well as pathological conditions. Importantly, we find that inhibition of TRPA1 prevents loss of CAPs during oxygen and glucose deprivation (OGD) and improves the recovery. TRPA1 block was effective when applied before, during, or after OGD, indicating that the TRPA1-mediated damage is occurring during both ischaemia and recovery, but importantly, that therapeutic intervention is possible after the ischaemic insult. These results indicate that TRPA1 has an important role in the brain, and that its block may be effective in treating many white matter diseases.

## 1. Introduction

Oligodendrocytes (OLs) wrap fatty myelin sheaths around axons to decrease the capacitance across the axonal membrane and increase the action potential speed. Myelin loss in diseases such as periventricular leukomalacia, leukodystrophies, multiple sclerosis, and stroke, leads to failure of neuronal transmission and thus mental and physical impairment. We have recently shown that cerebellar oligodendrocytes express transient receptor potential ankyrin-1 (TRPA1), whose activation during ischaemia causes excessive Ca^2+^ influx and myelin damage [1].

TRPA1 is one of a large family of tetrameric non-selective cation channels that are widely expressed in the grey and white matter of the CNS and are increasingly considered as potential therapeutic targets in brain disorders [2]. TRPA1 is sensitive to environmental irritants and endogenous electrophilic compounds that are formed during oxidative stress [3], which have been shown to evoke pain, cold, itch, and inflammation [4]. These compounds can activate TRPA1 by covalent modification of cysteine or lysine residues at distinct regions on its intracellular N-terminal; however, TRPA1 can also be activated by several other agonists that interact with regions within the pore. Like many TRP channels, TRPA1 is highly permeable to Na^+^ and Ca^2+^, which in turn can deregulate cell function and cause apoptosis. In cerebellar OLs, TRPA1 was shown to be activated during ischaemia via acidification of the cytosol [1]. However, until now we did not know whether OLs in other areas of the brain express functional TRPA1 and whether TRPA1 block is protective against loss of white matter function in ischaemia.

Here, we show by patch-clamping corpus callosum OLs, that they also respond to TRPA1 agonists by raising their intracellular Ca^2+^ concentrations ([Ca^2+^]_i_), and that TRPA1 inhibition with A-967079 (20 µM) reduces the resting [Ca^2+^]_i_. Furthermore, the TRPA1 antagonist also increases the amplitude of optic nerve compound action potential (CAP) recordings under normal physiological conditions and prevents a large proportion of the oxygen and glucose deprivation (OGD) -induced loss of action potential. This shows that TRPA1 may play a role in regulating neuronal excitability and TRPA1 inhibition may be a possible treatment for white matter damage in diseases where acute or chronic ischaemia has been implicated.

## 2. Results

### 2.1. Oligodendrocytes Express Tonically Active TRPA1

**Figure 1:**
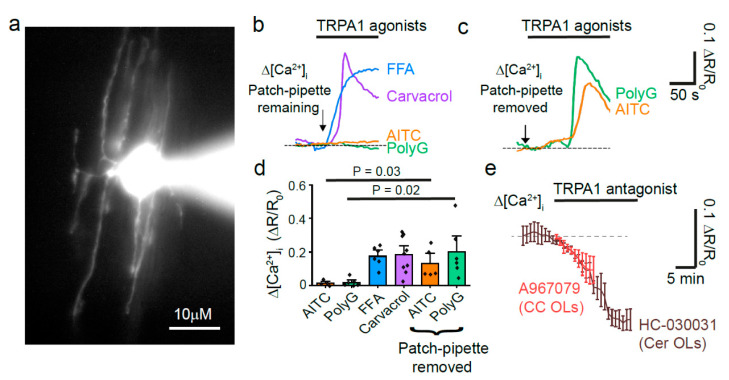
Corpus callosum oligodendrocytes express TRPA1. (**a**) High magnification of a whole-cell patch clamped OL in corpus callosum (CC) with Alexa dye in the soma and processes. (**b**) Representative Δ [Ca^2+^]_i_ trace responses to TRPA1 agonists with the patch-pipette remaining: FFA 100 µM (*n* = 6); carvacrol 2mM (*n* = 8); 500 µM AITC (*n* = 4); polygodial 100 µM (*n* = 6). (**c**) Δ [Ca^2+^]_i_ to TRPA1 agonists with the patch-pipette removed: AITC 500 µM (*n* = 5); polygodial 100 µM (*n* = 6). (**d**) Mean ± SEM maximum Δ [Ca^2+^]_i_ in response to TRPA1 agonists (*p*-values compared with and without patch pipette, one-way ANOVA test with Holm-Bonferroni correction for multiple comparisons). (**e**) The graph shows a decrease in resting [Ca^2+^]_i_ that occurs in the presence of TRPA1 antagonists: A-967079 20 µM in CC Ols and HC-030031 100 µM in cerebellar (Cer) OLs.

The corpus callosum (CC) is a white matter tract spanning across the two hemispheres that is often thinned or demyelinated as a result of local hypoperfusion occurring in periventricular leukomalacia or stroke victims [1]. Therefore, our first aim was to determine whether OLs in the CC also express functional TRPA1. To do this, we whole cell patch-clamped OLs identified at first by their morphology (light oval somata laid in lines within parallel axons) and then by their dye-filled morphology (*n* = 28, Figure 1a). Using the patch-pipette, the OLs were dye filled with the Ca^2+^ sensitive dye Fluo-8 (200 µM). This method ensures that the measured [Ca^2+^]_i_ changes are in OLs only. As previously reported in cerebellar OLs [2], the TRPA1 agonists flufenamic acid (FFA, 100 µM) and carvacrol (2 mM), evoke a [Ca^2+^]_i_ increase in OLs in the corpus callosum (P12-17; Figure 1b,d). However, while the patch pipette was present, the TRPA1 agonists AITC (500 µM) and polygodial (100 µM) did not evoke a calcium increase. When we repeated the experiments with the patch-pipette removed, a short while after loading the cell with the Ca^2+^ indicators, we then found that AITC and polygodial were able to activate TRPA1 (Figure 1c,d). This lack of response to the electrophilic, covalently binding TRPA1 agonists (AITC and polygodial, Table 1) during patch-clamping techniques has been found previously [3] and suggests that a necessary intracellular component may be washed out or chelated in these instances, thus modifying the availability of the agonist binding site. Importantly, application of the TRPA1 antagonists A-967079 (20 µM) or HC-030031 (100 µM), whose mechanisms of action do not involve the N-terminal, decreases the resting [Ca^2+^]_i_ in both corpus callosum and cerebellar OLs, as it does in astrocytes [4], suggesting that TRPA1 in OLs is tonically active (Figure 1e) and regulates normal cell functions.

### 2.2. Tonic TRPA1 Activation Regulates the Compound Action Potential Amplitude in the Optic Nerve

**Figure 2:**
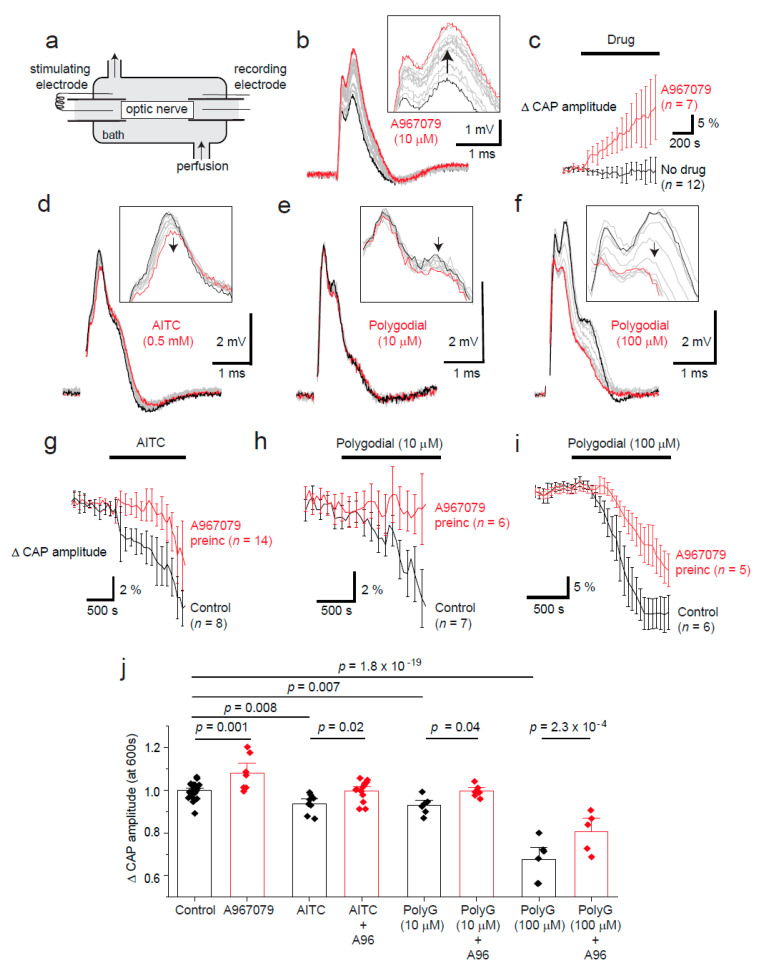
TRPA1 agonist and antagonist effects on optic nerve compound action potential amplitude. (**a**) Compound action potentials (CAPs) were measured in young adult optic nerves (P42–P70). (**b**) A-967079 (A96, 10 μM) block of TRPA1 increases the CAP amplitude (black trace at start; with time this increases (grey traces), and the red trace is the final trace). The arrows indicate the direction of change in CAP measurements with time. (**c**) Mean (± SEM) CAP amplitude increases after application of A-967079 (10 μM; *n* = 7) compared to vehicle (DMSO; *n* = 12), and decreases after TRPA1 agonist application: AITC (500 μM, (**d**,**g**,**j**), *n* =8); and polygodial (10 μM, (**e**,**h**,**j**), *n* = 7; and 100 μM, (**f**,**i**,**j**), *n* = 6). TRPA1–evoked decreases can be significantly inhibited by over 15 min of preincubation with A-967079 (20 μM, **g**–**j**). (**j**) Bar graph showing the mean (± SEM) changes in CAP amplitude (each *n* is depicted as a diamond data point) after application of agonists and antagonists for 10 min. *p*-values are from multiple comparisons using a one-way ANOVA with Holm–Bonferroni correction.

White matter Ca^2+^ concentrations, and receptors that allow Ca^2+^ flux, have been suggested to regulate action potential propagation in disease [12,13,14,15,16]. As TRPA1 regulates intracellular Ca^2+^ concentrations in oligodendrocytes, we hypothesised that tonic TRPA1 activity may influence oligodendrocyte function in maintaining fast neuronal action potential propagation. We used the optic nerve to measure CAP amplitude as described previously ([15,17]; Figure 2a) because it is a heavily myelinated, easily accessible CNS white matter tract [13]. Interestingly, we discovered a modest regulation of CAP amplitude by tonic TRPA1 activity in the optic nerve as TRPA1 inhibition with A-967079 (20 µM) increased the amplitude by 12 ± 4% within 10 min (*n* = 9; Figure 2b,c). Conversely, the TRPA1 agonists AITC (500 µM; *n* = 8; Figure 2d,g), and polygodial (10 or 100 µM; *n* = 6–7; Figure 2e,f,h,i) reduced the CAP amplitude (*n* = 5–14; Figure 2j). The TRPA1 agonist-induced decrease in compound action potential amplitude was reduced in the presence of A-967079, which was preincubated for over 15 min to allow for the initial increase in CAP amplitude before application of the agonists (*n* = 5–14; Figure 2j). As optic nerves do not include neuronal somata, these results indicate glial TRPA1 regulates retinal ganglion cell CAP propagation in physiological conditions. Whether this is due to OL or astrocyte TRPA1 is yet to be determined.

### 2.3. TRPA1 Block Protects against Loss of the Compound Action Potential during OGD

**Figure 3:**
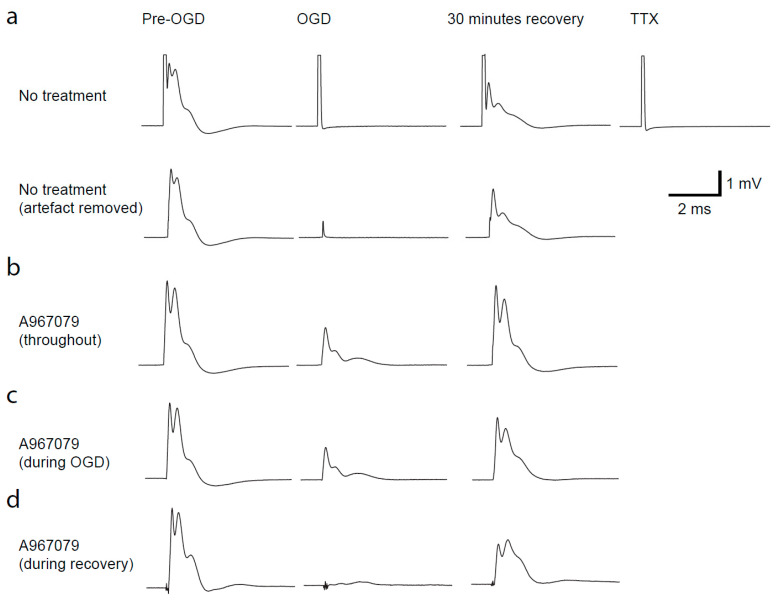
Ischaemia-evoked changes in optic nerve compound action potential (CAP). (**a**) Top row, traces from the left are: CAP in control solution, after 30 min ischemia, after 30 min recovery to control solution, after application of 500 nM TTX to record the stimulus artefact. Row b shows the same traces after the stimulus artefact has been removed from the traces. (**b**–**d**) Artefact subtracted responses with A-967079 (20 μM) preincubated and added throughout the experiment (**b**) during ischaemia only (**c**) or during the recovery only (**d**).

**Figure 4:**
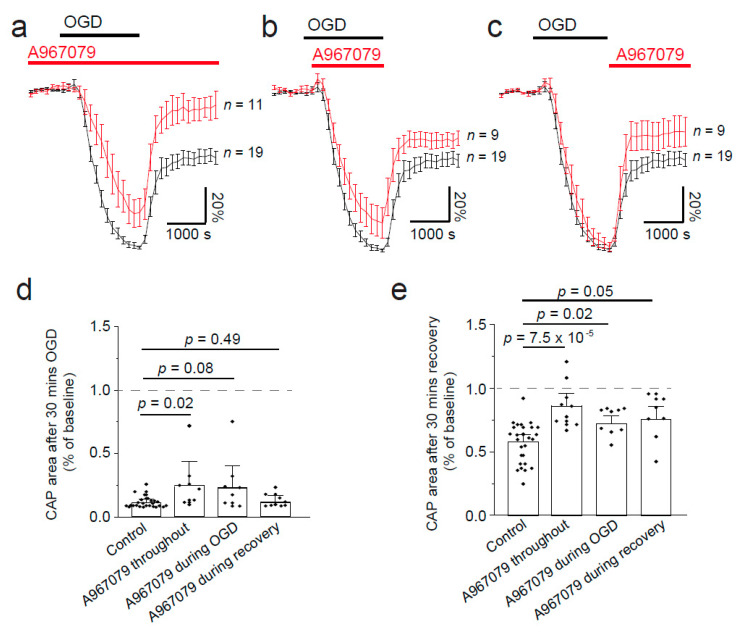
Ischaemia-evoked changes in optic nerve compound action potential (CAP) are reduced by TRPA1 block with A-967079. (**a**) Normalised area of CAP before, during, and after ischaemia, simulated by removing oxygen and glucose and replacing them with nitrogen and sucrose, showing how preincubation of the TRPA1 antagonist (A-967079, 20 μM) can prevent the loss of the CAP during ischaemia and improve the recovery after replacement of oxygen and glucose. Application of A-967079 during ischaemia (**b**) or during recovery (**c**) only is also protective. (**d**) Normalised CAP area after 30 min in OGD or after 30 min recovery (**e**). *p*-values in (**d**) are from Mann–Whitney tests with Holm–Bonferroni correction for multiple comparisons. *p*-values in (**e**) are from a one-way ANOVA with Holm–Bonferroni correction for multiple comparisons.

In the past, CAP recordings have demonstrated that simulated ischaemia results in a reduction in action potential propagation, with only a partial recovery [14,15,18,19,20]. This reduction in CAP amplitude is thought to be mediated by Ca^2+^ influx through different channels; therefore, TRPA1 inhibition may protect against loss of CAPs during ischaemia. To simulate ischaemia we removed O_2_ and glucose for 30 min, and continually recorded the CAP during a recovery period of 30 min (Figure 3). The area under the curve of the CAP was measured throughout. To understand the time at which TRPA1 activation may cause the most damage, we applied A-967079 at different times during the experiment. Firstly, we preincubated A-967079 (20 µM) and administered it throughout the experiment and found it significantly reduced the loss of the CAP during ischaemia (Figure 3b, and Figure 4a,d; *p* = 0.02) and improved the recovery from 61 ± 3.7% to 89 ± 7.4% (Figure 4a,e; *p* = 7.5 × 10^−5^). As therapeutics are more often than not given either during or after the event rather than prophylactically, we applied A-967079 during either the ischaemic insult or only the recovery period (Figure 3c,d and Figure 4b–e). A-967079 application during the ischaemic insult or the recovery period alone resulted in an improved CAP recovery (77 ± 8%; *p* = 0.02 and 73.7 ± 4%; *p* = 0.05, respectively) suggesting that the TRPA1-mediated damage is occurring during both the ischaemic and recovery periods. These results suggest that TRPA1 activation during ischaemia is a major cause of white matter damage and that TRPA1 antagonists may be effective in treating white matter in the many diseases involving ischaemia.

## 3. Discussion

The presence of TRPA1 in glial cells is a new concept, but the evidence for functional TRPA1 in both astrocytes [4,21] and OLs is growing [2,22,23,24,25], reviewed in [26]. In astrocytes and OLs, TRPA1 activation and inhibition raises and decreases basal intracellular Ca^2+^ (and Mg^2+^) concentrations, respectively [2,21] (Figure 1e). Astrocyte TRPA1 regulates neuronal function [4,21], and TRPA1 knockout appears to modify the expression of myelinating proteins [22]. Here, we add to this evidence by showing that TRPA1 expression in OLs is conserved in other CNS brain areas, and that activation of TRPA1 in corpus callosum OLs generates a substantial Ca^2+^ influx. Despite this, we did not find a large TRPA1-mediated non-specific cation current (that would reverse at −10 to 0 mV) as you would expect when TRPA1 is activated in large numbers on the cells [27]. Instead, we saw a TRPA1-mediated decrease in potassium conductance indicating a TRPA1-mediated inhibition of potassium channels. This has already been shown to occur when TRPA1 is activated during ischaemia [2]. This suggests that the levels of TRPA1 in OLs are low, which may be why its expression is not picked up in bulk brain studies [28], but is in single cell sequencing [23] and in situ hybridisation experiments [2]. Interestingly, TRPA1 was not found in mouse optic nerves during qPCR experiments [29]. This discrepancy is hard to explain as the mouse ages and strain were similar to those used here (C57BL6; P7, P30-40 vs. P12-18 and P42-70 here). Nonetheless, our evidence clearly points to functional expression of TRPA1 in the mouse optic nerve, and we provide the first evidence for a role for glial TRPA1 in regulating neuronal transmission through the white matter. Although the focus of this paper was the expression of TRPA1 in OLs, the effect of TRPA1 on astrocytes and possibly oligodendrocyte progenitor cells may be equally important. Future investigations will aim to specifically assess oligodendrocyte TRPA1 activity through use of TRPA1-shRNA or conditional knockouts.

How TRPA1 activity decreases the size of the CAPs under physiological conditions has not been completely elucidated. We know that removing extracellular Ca^2+^ protects against loss of CAPs during ischaemia [14], suggesting that influx of Ca^2+^ through TRPA1 may diminish the CAP. However, we also know that TRPA1 decreases oligodendrocyte potassium permeability ([2], Giacco et al., unpublished), which would in turn decrease the CAP amplitude by decreasing glial cell potassium syphoning away from the periaxonal space [30,31]. How the CAP is affected by inhibited OL potassium conductance is dependent on the amount that this changes the perinodal potassium concentration. Changes of a few mM can depolarise axons and lead to an increased excitability, but large changes (>10 mM) can lead to conduction block [32,33]. In our model, it appears that tonic TRPA1 activation in normal physiological conditions is already limiting axon excitability, or causing conduction block of a small set of axons and increased TRPA1 activation with exogenously applied agonists that builds upon that phenomenon. Therefore, it appears that either perinodal potassium concentrations are already high enough to prevent action propagation in some axons, or that the OL depolarisation or Ca^2+^ influx through TRPA1 is decreasing the excitability of axons in another way. In support of the former, one action potential is thought to increase the perinodal K^+^ concentration by 1 mM [34], and this may increase quickly with successive action potentials if potassium syphoning is hindered.

As mentioned above, increased extracellular Ca^2+^ concentrations are correlated with the amount of white matter damage occurring during ischaemia [12,14,35], and a large proportion of the Ca^2+^-mediated damage has long been thought to be due to glutamate excitotoxicity driving Ca^2+^ influx through OL AMPA/KA [35,36,37,38] and NMDA [12,14,15,39,40,41,42] receptors. However, our recent work indicates that the majority of the Ca^2+^ influx into OLs during ischaemia is through TRPA1, which becomes activated when OLs acidify as a result of raised extracellular potassium concentrations occurring during ischaemia [2]. Optic nerves subjected to ischaemia for 1 hour have TRPA1-mediated myelin damage shown with electron microscopy [2]. In line with that, we find here that TRPA1 inhibition is beneficial at protecting against white matter damage when applied during, after, or throughout the ischaemic insult, suggesting that TRPA1 block may be used as a potential prophylactic therapy, or one to improve recovery after a stroke. It is important to add, however, that the CAP area never returned to its pre-ischaemic level. This residual loss of the CAP may be due to activation of glutamate receptors, or due to underlying damage to the axons [36], rather than OLs.

At present, there are a large number of patents for the use of TRPA1 antagonists in human pathologies. A-967079 was chosen here because it is commercially available, highly selective, more readily dissolved in aqueous solution than HC-030031, and can penetrate the nervous system at therapeutic concentrations when administered peripherally, by oral gavage and intraperitoneal injection [43]. This opens up possibilities for testing the effects of A-967079 in vivo in stroke models. However, initial evidence suggests that TRPA1 activation on capillaries may induce vasodilation of arterioles and protect against widespread damage during stroke [44,45]. These results were determined using a conditional endothelial cell TRPA1 knockout [46]. Nonetheless, the evidence of TRPA1-mediated pathology within the parenchyma is growing substantially and indicates that targeted TRPA1 knockout or block within the parenchyma will have major benefits and needs investigating further.

## 4. Materials and Methods

### 4.1. Animals

C57BL/6J mice of either sex were killed via schedule 1 (cervical dislocation) in accordance with the guidelines of the UK Animals (Scientific Procedures) Act 1986 and subsequent amendments. The protocols were approved by the Animal Welfare Ethical Review Body of Guy’s Campus, King’s College London (PPL number P7322191B, granted on 26 April 2019 and amended on 11 November 2019).

### 4.2. Brain Slice and Optic Nerve Preparation

Coronal brain slices (225 µm thick) were prepared from the brains of P12-P17 mice in ice-cold solution containing (mM) 124 NaCl, 26 NaHCO_3_, 1 NaH2PO4, 2.5 KCl, 2 MgCl_2_, 2 CaCl_2_, 10 glucose, bubbled with 95% O_2_/5% CO_2_, pH 7.4, as well as 1 mM Na-kynurenate to block glutamate receptors. Optic nerves were dissected from P42-P70 mice. Brain slices and optic nerves were then incubated at room temperature (21–24 °C) in the above solution until used in experiments.

### 4.3. Cell Identification and Electrophysiology

Oligodendrocytes were identified by their location and morphology. All cells were whole-cell clamped with pipettes with a series resistance of 8–35 MΩ. Electrode junction potentials were compensated, and cells were voltage-clamped at −74 mV.

### 4.4. External Solutions

Slices and optic nerves were superfused with bicarbonate-buffered solution containing (mM) 124 NaCl, 2.5 KCl, 26 NaHCO_3_, 1 NaH_2_PO_4_, 2 CaCl_2_, 1 MgCl_2_, 10 glucose, pH 7.4, bubbled with 95% O_2_ and 5% CO_2_. Applications of TRPA1 channel agonists and antagonists to brain slices were at room temperature. All CAP recording and OGD experiments were performed between 33 °C and 36 °C. Data were excluded if changes in temperature were observed during the experiment. Control and drug conditions were interleaved and the experimenter was blinded to the solution contents. To simulate ischaemia, we replaced external O_2_ with N_2_, and external glucose with 7 mM sucrose. The flow rate was approximately 4 mL/min into a 1.5 mL bath, giving a turnover rate of under 25 s.

### 4.5. Intracellular Solutions

Cells were whole-cell clamped with electrodes containing K-gluconate-based solution, comprising (mM) 130 K-gluconate, 2 NaCl, 0.5 CaCl_2_, 10 HEPES, 10 BAPTA, 2 NaATP, 0.5 Na_2_GTP, 2 MgCl, and 0.05 Alexa Fluor 594, and pH set to 7.15 with K-OH (all from Sigma-Aldrich, Germany). For Ca^2+^ imaging experiments, BAPTA was decreased to 0.01 mM and replaced with 10 mM phosphocreatine, CaCl_2_ was reduced to 10 µM, and 200 µM Fluo-4 or Fluo-8 was added to allow ratiometric imaging with the above Alexa Fluor.

### 4.6. Single Cell Ion Imaging

Fluo-8 and Alexa Fluor 594 were used in the internal solution to measure [Ca^2+^]_i_ changes ratiometrically during experiments. Fluo-8 and Alexa Fluor 594 fluorescence were excited sequentially using a monochromator every 3 s at 488 ± 10 nm and 585 ± 10 nm, and emission was collected using a triband filter cube (DAPI/FITC/Texas Red, 69002, Chroma Technology Corporation, Bellow Falls, VT, USA). The mean ratio of intensities (excited at 488 nm/excited at 585 nm) before applying the TRPA1 agonist was 1.010 (*n* = 28).

### 4.7. Drug Application

Stock solutions of the following drugs were made up: Carvacrol (Sigma-Aldrich, Darmstadt, Germany) was diluted in 100% ethanol, HC 030031 (Tocris Bioscience, Bristol, UK), A-967079 (Boc Sciences, Shirley, NY, USA), flufenamic acid (Sigma-Aldrich, Darmstadt, Germany), polygodial (Tocris Bioscience, Bristol, UK), and AITC (Sigma-Aldrich, Darmstadt, Germany) were made up in 100% DMSO. For CAP experiments, DMSO and ethanol were also added to control solutions at the same concentrations and did not evoke any changes in the CAP amplitude at the concentrations used (≤0.1%). For the patch-clamping experiments, vehicle controls were tested on oligodendrocytes and did not evoke any changes at the concentrations used. Stocks were kept at −20 °C apart from carvacrol and AITC, which were made up fresh on each day of use. To minimise evaporation of carvacrol, lids were kept on until the solutions were used. The control and test experiments were interleaved, and for all CAP recordings, the experimenter was blinded to the contents of the solutions.

### 4.8. Compound Action Potential Recording

Optic nerves were isolated from P42-P70 mice, and CAP recorded using suction electrodes [13]. The optic nerve was stimulated using a suction electrode filled with extracellular solution, using 0.2 ms, 50–100 V pulses, at 1 Hz to produce as supramaximal a response as possible. Supramaximal stimulation (40% over the voltage producing the maximal amplitude of CAP) was constrained by a maximum stimulation voltage of 100 V. The CAP was recorded as a voltage using an Axon Axoclamp 2B amplifier, with the recording suction electrodes placed as far away from the stimulating electrode as possible to obtain a CAP waveform with three peaks, which denoted heavily myelinated axons (peak 1), normally myelinated axons (peak 2) and unmyelinated axons (peak 3; [9]). At the end of each experiment, TTX (500 nM) was applied to obtain the stimulus artefact in the absence of action potentials, which were subtracted from all the records obtained previously. The pre-stimulus baseline of the resulting traces was subtracted, then the traces were rectified (squared, then square rooted), and either the area of the CAP was calculated or the amplitude of the second peak was measured (see results).

### 4.9. Statistics

Data were mean ± s.e.m. *p*-values were from ANOVA tests (for normally distributed data) and Mann–Whitney U or Kruskal–Wallis tests (for non-normally distributed data). Normally distributed data were tested for equal variance (*p* < 0.05, unpaired F-test), and paired *t*-Tests were adjusted accordingly. *p*-values quoted in the text were from ANOVA tests unless otherwise stated. For multiple comparisons, *p*-values were corrected using the Holm–Bonferroni or Tukey’s test. Small sample sizes were often able to achieve statistical significance, so in those instances, power analysis of sample size was also measured for soma data and determined to be greater than 0.75. Data normality was assessed using Shapiro–Wilk tests. All statistical analysis was conducted using OriginLab or GraphPad Prism software.

## 5. Conclusions

We provided the first evidence that TRPA1 activation in the white matter suppresses axonal function in both health and disease. Whether depression of neuronal activity is important for normal brain function is not known, but our results strongly indicate that TRPA1 block may preserve white matter function during and after ischaemic episodes in the brain. In light of the evidence provided above, it will be important to dissect the role of TRPA1 expressed by the different cell types to enhance our understanding of how the CNS is affected by TRPA1 activity in the brain.

## Figures and Tables

**Table 1:** TRPA1 agonist binding sites.

TRPA1 Agonist	Covalent Binding (N-Terminal)	Non-Covalent Binding
Carvacrol	-	Unknown other site [5]
Flufenamic Acid (FFA)	-	Unknown other site [6]
Menthol	-	TM5 [7]
Isoflurane, propofol	-	TM5, TM6 and the pore helix 1 [8]
A-967079 (antagonist)	-	TM5, TM6 and the pore helix 1 [8]
Polygodial (PolyG)	Lys residues [9]	-
AITC (mustard oil)	Cys and Lys residues [10]	-
Cinnemaldehyde	Cys and Lys residues [10]	-
JT010	Cys621 only [11]	-

## Data Availability

Please contact the corresponding author to obtain the original data which, for the electrophysiology data, are in the form of abf files that can be read by free Clampfit Software (Molecular Devices). Imaging data are available as files composed by the MetaFluor Imaging Series that can be imported for analysis into ImageJ.
